# Correlation between the body roundness index and chronic obstructive pulmonary disease: a cross-sectional analysis

**DOI:** 10.3389/fnut.2025.1624617

**Published:** 2025-08-25

**Authors:** Longqian Li, Mingzhi Lin, ZhuoLin Qin, Yan Liu, Xiangji Dang, Cheng Wang

**Affiliations:** ^1^The Second Clinical Medical College, Lanzhou University, Lanzhou, China; ^2^School of Pharmacy, Lanzhou University, Lanzhou, China; ^3^Department of Pharmacy, Lanzhou University Second Hospital, Lanzhou, China; ^4^Department of Thoracic Surgery, Lanzhou University Second Hospital, Lanzhou, China

**Keywords:** chronic obstructive pulmonary disease (COPD), body roundness index (BRI), the National Health and Nutrition Examination Survey (NHANES), abdominal fat, metabolism

## Abstract

**Background:**

Chronic obstructive pulmonary disease (COPD) is a leading cause of death worldwide, with abdominal fat, particularly visceral fat, closely associated with its onset and progression. While the lipid accumulation product (LAP) has been linked to COPD risk, it is not sufficient to fully reflect the level of visceral fat. In contrast, the body roundness index (BRI), a more accurate measure of abdominal fat distribution, has not been fully explored in relation to COPD.

**Methods:**

The study used data from the National Health and Nutrition Examination Survey (NHANES) from 1999 to 2018 for a cross-sectional analysis, including 24,873 participants aged over 40 years. Multivariate logistic regression models evaluated the relationship between BRI and COPD. Subsequently, sensitivity analysis, subgroup analysis, restricted cubic spline (RCS) modeling, and threshold effect evaluation were conducted.

**Results:**

A multivariate logistic regression analysis showed that, for every 1-unit increase in the BRI, the risk of COPD increased by 5.1% (95% CI: 1.022–1.080; *p* < 0.001). Sensitivity analysis confirmed this conclusion (AUC_BRL_, 0.78745 and AUC _LAP_, 0.78675). In addition, the BRI is significantly better than the LAP (OR = 1.001, 95% CI: 1.000–1.001, *p* = 0.202) in predicting COPD. The restricted cubic spline (RCS) analysis showed a “U-shaped” relationship between the BRI and COPD, and the threshold effect analysis determined the critical point of BRI to be 3.6583.

**Conclusion:**

This study demonstrates that the body roundness index (BRI) is significantly associated with COPD risk, with a threshold effect observed at a BRI value of 3.6583. A 1-unit increase in the BRI corresponds to a 5.1% higher COPD risk. The BRI proves to be a more accurate indicator of abdominal fat distribution compared to traditional measures such as the lipid accumulation product (LAP), making it a useful tool for early COPD risk assessment and intervention.

## 1 Introduction

Chronic obstructive pulmonary disease (COPD) is one of the most prevalent chronic respiratory diseases globally, affecting approximately 10% of the population. It is characterized by persistent and usually irreversible airflow limitation (1). Due to the increasing global aging population, urbanization, and the rising associated risk factors, chronic obstructive pulmonary disease (COPD) has emerged as one of the leading causes of mortality on a global scale (2). According to predictions by the World Health Organization (WHO), COPD will be the fourth leading cause of death globally by 2060, with its prevalence expected to continue rising. Statistics show that, by 2060, over 5.4 million deaths annually may be attributed to COPD and its related complications (3–5).

Studies have shown that COPD is closely associated with age (6). As individuals age, lung function gradually declines, especially under the influence of smoking or other environmental factors, significantly increasing the risk of developing COPD. Age has been identified as an independent risk factor for COPD progression, with elderly individuals often exhibiting more severe symptoms and higher risks of comorbidities (6, 7). Particularly, the prevalence and severity of COPD increase significantly in individuals aged 40 and above (8). For instance, a large-scale study conducted in China revealed that the COPD prevalence in individuals aged 40 and above was 13.7%, which was significantly higher than the 2.1% prevalence in those aged 20–39 years (9). Furthermore, another study in Australia found that the COPD prevalence in individuals aged 40 years and above was 7.5%, and the prevalence in those aged 75 years and above was 29.2% (10). As age progresses, lung structure and function deteriorate, with pathological changes such as emphysema and airway remodeling intensifying, ultimately leading to continuous worsening of respiratory function and airflow limitation (11). Additionally, COPD patients often exhibit accelerated aging, with lung deterioration occurring faster than the expected biological aging process (12). Oxidative stress and chronic inflammation are key factors in accelerating aging, exacerbating cellular damage, and inhibiting repair mechanisms, further accelerating lung tissue aging (13). Furthermore, elderly individuals with COPD often have more comorbidities, such as cardiovascular diseases and metabolic disorders, which complicate treatment and worsen prognosis (2).

In addition to age and environmental factors, obesity, particularly the accumulation of visceral fat, has been identified as a major factor influencing the development and progression of COPD. Obesity promotes COPD progression through various mechanisms, including inducing adipocytes to secrete pro-inflammatory cytokines such as tumor necrosis factor-*α* (TNF-α) and interleukin-6 (IL-6), thereby increasing systemic inflammation, which exacerbates airway remodeling and lung function deterioration (6, 14). Visceral fat further exacerbates airway remodeling and lung function deterioration by secreting TNF-*α* and IL-6, leading to systemic inflammation (6, 7). Existing studies have shown that lipid accumulation products (LAP) are associated with the development of COPD (4, 6). However, although LAP has been shown to effectively assess visceral fat and its close association with various diseases, its application in COPD risk prediction remains limited, as it primarily focuses on waist circumference (WC) and does not fully consider other aspects of body shape and fat distribution. Previous studies have shown that the accumulation of visceral fat is closely associated with the development of COPD, and the degree of its accumulation can reflect an individual’s metabolic risk. Therefore, a quantitative approach for assessing fat distribution and disease progression in COPD patients is needed to provide a more accurate foundation for early intervention and personalized treatment. The body roundness index (BRI), recognized as a novel evaluation tool, provides a more precise representation of visceral fat distribution. The BRI quantifies the “roundness” of the body by combining waist circumference and height, providing a more comprehensive method for assessing abdominal fat accumulation (15, 16).

This study aimed to investigate the relationship between the BRI and COPD, with a further examination of their potential association by utilizing data from the National Health and Nutrition Examination Survey (NHANES). NHANES uses a complex sampling method combined with a multi-stage stratified design to accurately represent the health status of the non-institutionalized American population. This study facilitates a comprehensive analysis of this connection within a diverse population in the United States. Furthermore, it aims to provide valuable insights pertinent to clinical practice and to advance the development of enhanced strategies for the prediction and intervention of COPD.

## 2 Methods

### 2.1 Study design and sample

In this cross-sectional analysis, we used data from the NHANES (1999–2018) to explore the relationship between the BRI and COPD. All participants provided written informed consent before joining the survey, and this study was conducted under ethical guidelines approved by the relevant review committee.

**Figure 1:**
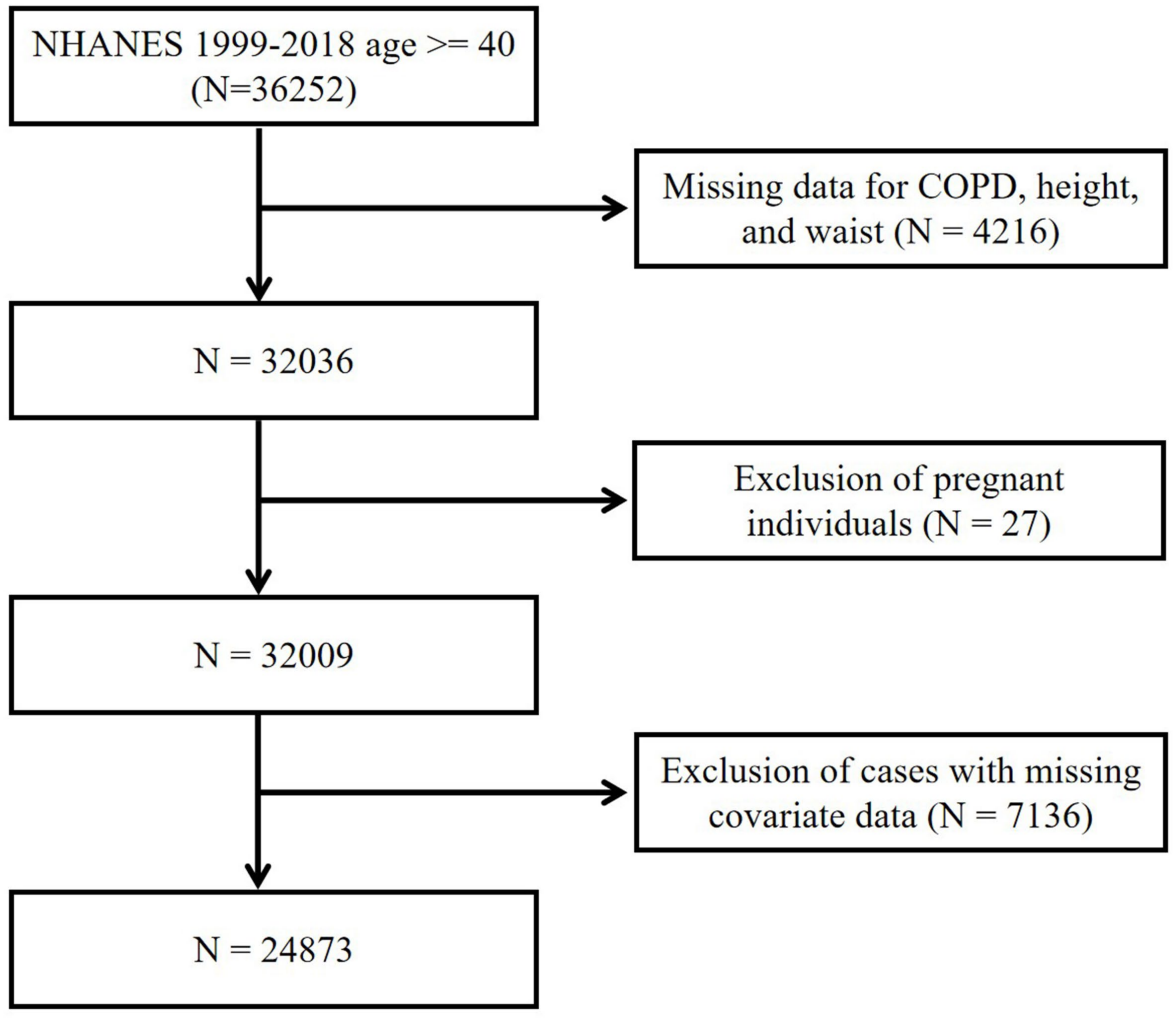
Flow diagram of the selection of eligible participants. NHANES, National Health and Nutrition Examination Survey; COPD, Chronic obstructive pulmonary disease.

This study included participants aged 40 years and older (*N* = 36,252). To ensure the integrity and validity of the study, we applied the following exclusion criteria: (1) age less than 40, (2) pregnancy, (3) missing BRI and COPD-related data, and (4) missing key covariates data. After applying these exclusion criteria, a total of 24,873 participants were finally included in the final analysis (Figure 1).

### 2.2 Assessment of COPD

In this study, the diagnosis of COPD was obtained through self-reported questionnaires completed by the participants. Participants were asked whether they had ever been diagnosed with COPD, emphysema, or chronic bronchitis by a doctor or healthcare professional. This questionnaire-based method effectively identifies COPD patients and has been widely used in related studies, demonstrating a certain degree of reliability (4).

### 2.3 Assessment of the BRI

The BRI is an indicator used to evaluate body shape and quantify the accumulation of abdominal fat. Unlike traditional body mass index (BMI), the BRI more accurately reflects the distribution of abdominal fat, especially the accumulation of visceral fat. The calculation formula for BRI is as follows (17):


$$\mathrm{BRI}=364.2-365.5\sqrt{1-{\left(\frac{\mathrm{WC}(m)÷2\pi }{0.5\text{height}\;(m)}\right)}^{2}}$$

### 2.4 Covariates

In this study, we included a wide range of potential covariates that may be associated with the occurrence of COPD to ensure the comprehensiveness and accuracy of the analysis. Demographic characteristics included age, sex, marital status (such as married, never married, divorced, widowed, separated, and living with a partner), and race (including non-Hispanic White, non-Hispanic Black, other Hispanic, Mexican American, and other races). Education level was categorized into several groups, including less than 9th grade, 9–11th grade, high school graduate/GED or equivalent, college graduate or above, and some college or AA degree. Additionally, lifestyle factors such as smoking status (current smokers, former smokers, and never smokers) and alcohol consumption (former, never, mild, moderate, and heavy drinkers) were also included.

In terms of health status, we incorporated variables such as the poverty-to-income ratio (PIR), weight, waist circumference, and the presence of conditions such as hypertension, diabetes mellitus (DM), cardiovascular disease (CVD), chronic kidney disease (CKD), and cancer. These variables serve as important determinants for assessing the risk of COPD (18, 19). Moreover, several key biochemical markers were included, including alanine aminotransferase (ALT), aspartate aminotransferase (AST), albumin, creatinine, uric acid, blood urea nitrogen (BUN), total cholesterol (refrige total cholesterol mg.dl), triglycerides, high-density lipoprotein (HDL), white blood cell (WBC), lymphocytes, monocytes (MO), segmented neutrophils (SE), eosinophils (EO), basophils (BA), red blood cell (RBC), hemoglobin, and platelets. These blood parameters are essential for understanding the role of visceral fat, metabolic status, and systemic inflammation in the context of COPD (20, 21).

### 2.5 Statistical analyses

This study used various statistical methods for a systematic analysis of the data. Descriptive statistical analysis was first performed on demographic characteristics. Continuous variables were expressed as mean ± SD and compared between groups using t-tests or Kruskal–Wallis rank sum tests. Categorical variables were presented as percentages and compared using chi-square tests. To ensure model stability and minimize the risk of multicollinearity, the variance inflation factor (VIF) was used to screen independent variables. VIF quantifies the level of collinearity among variables, and variables with a VIF greater than 5 were excluded to improve model stability and interpretability (22).

For further optimization of variable selection, Lasso regression was used. Lasso regression, with L1 regularization, shrinks the coefficients of less important variables to zero, achieving dimensionality reduction and enhancing the model’s generalizability, thereby preventing overfitting. Furthermore, to capture potential non-linear relationships between the BRI and COPD, restricted cubic spline (RCS) analysis was applied. A multivariable logistic regression analysis was performed to calculate odds ratios (ORs) and 95% confidence intervals (CIs) to assess the association between the BRI and COPD. The optimal BRI cutoff value was determined through threshold effect analysis, and a comparison was performed between the BRI, traditional BMI, and LAP. Additionally, sensitivity analysis was performed by excluding outliers below the 5th percentile and above the 95th percentile in the BRI. Subgroup analysis was also conducted to further explore the relationship between the BRI and COPD.

All statistical analyses were performed using R software (version 4.4.2).

## 3 Results

### 3.1 Baseline characteristics of participants

The study included a total of 24,873 participants, of which 1,499 were diagnosed with COPD (the COPD group) and 23,374 without COPD (the non-COPD group). The baseline demographic characteristics analysis shown in Table 1 revealed that the COPD group had a significantly higher average age (64.1 ± 11.6 years) compared to the non-COPD group (59.3 ± 12.4 years, *p* < 0.001). Additionally, the COPD group had a significantly lower poverty-to-income ratio (PIR) (2.3 ± 1.6, *p* < 0.001). In terms of WBC, the COPD group exhibited significantly higher levels (7.7 ± 2.7 × 10^3^/μL) compared to the non-COPD group (7.1 ± 3.7 × 10^3^/μL, *p* < 0.001). Moreover, the COPD group showed increased numbers of eosinophils, monocytes, and segmented neutrophils (*p* < 0.001). Hemoglobin levels did not differ significantly between the two groups (*p* = 0.007), but platelet count was significantly higher in the COPD group (*p* = 0.005). Regarding metabolic-related biochemical markers, the COPD group had slightly elevated serum uric acid (*p* = 0.006) and creatinine (*p* < 0.001) levels. The prevalence of hypertension (65.2%), diabetes (28.6%), and cardiovascular disease (32.0%) was notably higher in the COPD group (*p* < 0.001). In addition, the BRI in the COPD group (6.0) was significantly higher than that in the non-COPD group (5.7).

**Table 1:** Population characteristic analysis of NHANES participants from 1999 to 2018.

Characteristic	Non-COPD	COPD	*p*-value
*N*	23,374	1,499	
Age (year)	59.3 ± 12.4	64.1 ± 11.6	<0.001
PIR	2.7 ± 1.6	2.3 ± 1.6	<0.001
Height (m)	1.7 ± 0.1	1.7 ± 0.1	<0.001
Weight (kg)	81.7 ± 19.9	82.3 ± 21.6	0.537
WC (m)	1.0 ± 0.2	1.0 ± 0.2	<0.001
ALT (U/L)	25.2 ± 22.6	25.1 ± 39.3	<0.001
AST (U/L)	25.9 ± 19.2	26.6 ± 22.1	0.55
Bilirubin (mg.dl)	0.7 ± 0.3	0.7 ± 0.3	0.498
Albumin (g.L)	42.2 ± 3.1	41.5 ± 3.3	<0.001
Creatinine (mg.dl)	0.9 ± 0.5	1.0 ± 0.6	<0.001
Uric acid (mg.dl)	5.5 ± 1.4	5.7 ± 1.6	0.006
Blood urea nitrogen (mg.dl)	14.8 ± 6.3	15.0 ± 6.9	0.893
Refrige total cholesterol (mg.dl)	200.9 ± 42.2	194.4 ± 44.7	<0.001
Refrige triglycerides (mg.dl)	159.0 ± 119.7	159.6 ± 125.0	0.297
HDL cholesterol (mg.dl)	53.4 ± 16.4	53.4 ± 18.1	0.453
WBC (1000cells.ul)	7.1 ± 3.7	7.7 ± 2.7	<0.001
Lymphocyte number (1,000 cells.ul)	2.1 ± 2.8	2.1 ± 1.6	0.071
Monocyte number (1,000 cells.ul)	0.6 ± 0.2	0.6 ± 0.3	<0.001
Segmented neutrophil number (1,000 cells.ul)	4.2 ± 1.7	4.7 ± 1.9	<0.001
Eosinophil number (1,000 cells.ul)	0.2 ± 0.2	0.2 ± 0.2	<0.001
Basophil number (1,000 cells.ul)	0.0 ± 0.1	0.1 ± 0.1	<0.001
Red blood cell count (million cells.uL)	4.6 ± 0.5	4.6 ± 0.5	0.607
Platelet	247.9 ± 67.5	254.1 ± 75.2	0.005
BRI	5.7 ± 2.2	6.0 ± 2.4	<0.001
Sex			<0.001
Female	11,784 (50.4%)	649 (43.3%)	
Male	11,590 (49.6%)	850 (56.7%)	
Race			<0.001
Non-Hispanic White	11,220 (48.0%)	1,014 (67.6%)	
Non-Hispanic Black	4,622 (19.8%)	232 (15.5%)	
Other Hispanic	1829 (7.8%)	85 (5.7%)	
Mexican American	3,943 (16.9%)	84 (5.6%)	
Other races—including multi-racial	1760 (7.5%)	84 (5.6%)	
Marital			<0.001
Married	1,694 (7.2%)	91 (6.1%)	
Never married	14,064 (60.2%)	784 (52.3%)	
Divorced	3,161 (13.5%)	253 (16.9%)	
Widowed	2,689 (11.5%)	239 (15.9%)	
Separated	801 (3.4%)	64 (4.3%)	
Living with partner	965 (4.1%)	68 (4.5%)	
Education			<0.001
Less than 9th grade	3,153 (13.5%)	201 (13.4%)	
9–11th grade (includes 12th grade with no diploma)	3,241 (13.9%)	287 (19.1%)	
High school graduate/GED or equivalent	5,377 (23.0%)	369 (24.6%)	
College graduate or above	5,297 (22.7%)	233 (15.5%)	
Some college or AA degree	6,306 (27.0%)	409 (27.3%)	
Hypertension			<0.001
No	10,736 (45.9%)	522 (34.8%)	
Yes	12,638 (54.1%)	977 (65.2%)	
Smoke			<0.001
Now	4,130 (17.7%)	531 (35.4%)	
Former	12,182 (52.1%)	227 (15.1%)	
Never	7,062 (30.2%)	741 (49.4%)	
Diabetes mellitus			<0.001
No	17,950 (76.8%)	1,071 (71.4%)	
Yes	5,424 (23.2%)	428 (28.6%)	
Alcohol			<0.001
Former	3,470 (14.8%)	108 (7.2%)	
Never	4,920 (21.0%)	481 (32.1%)	
Mild	8,602 (36.8%)	505 (33.7%)	
Moderate	3,113 (13.3%)	196 (13.1%)	
Heavy	3,269 (14.0%)	209 (13.9%)	
CKD			<0.001
No	17,985 (76.9%)	1,038 (69.2%)	
Yes	5,389 (23.1%)	461 (30.8%)	
CVD			<0.001
No	20,023 (85.7%)	1,019 (68.0%)	
Yes	3,351 (14.3%)	480 (32.0%)	
Cancer			<0.001
No	20,500 (87.7%)	1,180 (78.7%)	
Yes	2,874 (12.3%)	319 (21.3%)	
Cotinine (ng.ml)			<0.001
	52.2 ± 125.8	110.2 ± 160.9	

PIR, poverty-to-income ratio; WC, waist circumference; CKD, chronic kidney disease; CVD, cardiovascular disease; BRI, body roundness index.

### 3.2 Lasso regression and variance inflation factor analysis

We selected 28 significant variables from Table 1 for VIF analysis to address potential multicollinearity among variables, including age, sex, race, marital status, PIR, education level, hypertension, smoking status, alcohol, DM, height, WC, CKD, CVD, ALT, albumin, creatinine, uric acid, total cholesterol, WBC, Mo, Se, Eo, Ba, cancer, platelets, cotinine (cotinine ng.ml), and BRI. The VIF analysis results (Table 2) showed that, after excluding height, WC, and WBC, all remaining variables had a VIF value less than 5, indicating no multicollinearity issue.

**Table 2:** Variance inflation factor.

Variables	VIF1	VIF2	VIF3
Age	1.770	1.653	1.640
Sex	2.467	1.448	1.434
Race	1.225	1.152	1.149
Marital	1.075	1.075	1.074
PIR	1.251	1.238	1.237
Education	1.211	1.199	1.199
Hypertension	1.195	1.189	1.188
Smoke	1.111	1.109	1.109
Alcohol	1.180	1.180	1.178
Diabetes mellitus	1.216	1.209	1.208
Height	9.469	NA	NA
WC	51.276	NA	NA
CKD	1.401	1.399	1.399
CVD	1.196	1.195	1.194
ALT(U/L)	1.022	1.022	1.022
Albumin (g.L)	1.161	1.156	1.154
Creatinine (mg.dl)	1.301	1.296	1.296
Uric acid (mg.dl)	1.321	1.311	1.310
Refrige total cholesterol (mg.dl)	1.149	1.144	1.143
WBC (1,000 cells.ul)	5.363	5.402	NA
Monocyte number (1,000 cells.ul)	1.605	1.597	1.159
Segmented neutrophil number (1,000 cells.ul)	3.977	4.016	1.307
Eosinophil number (1,000 cells.ul)	1.169	1.173	1.161
Basophil number (1,000 cells.ul)	1.235	1.239	1.259
Cancer	1.104	1.103	1.104
Platelet	1.209	1.207	1.202
Cotinine (ng.ml)	1.241	1.236	1.227
BRI	51.203	1.331	1.328

PIR, poverty-to-income ratio; WC, waist circumference; CKD, chronic kidney disease; CVD, cardiovascular disease; BRI, body roundness index.

**Figure 2:**
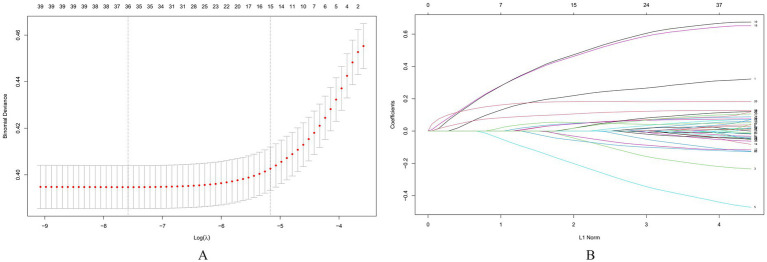
Screening of variables based on lasso regression. This figure illustrates the process of optimizing variable selection using Lasso regression. In 5-fold cross-validation, alpha = 1 is used for L1 regularization, and the optimal regularization parameter *λ* = 0.0005079675 is determined by minimizing the cross-validation error. This parameter optimized the performance of the model, and through the λ value, Lasso regression screened out 25 significant variables, which were included in the final logistic regression model for further analysis of their relationship with COPD. **(A)** The process of selecting the optimal λ value through cross-validation in the Lasso regression model, **(B)** The characteristics of the regression coefficients of each variable as a function of L1 regularization intensity.

Subsequently, we used Lasso regression for variable selection optimization, with alpha = 1 to perform standard L1 regularization. Lasso regression penalizes the model coefficients, driving the coefficients of less important variables to zero, thus achieving feature selection and dimensionality reduction. This method helps reduce model complexity and prevents overfitting. During model optimization, the best *λ* value (λ_min_ = 0.0005079675) was determined through 5-fold cross-validation, and the model was retrained using this λ value. Ultimately, 25 variables were selected and included in the logistic regression model (as shown in Figure 2).

### 3.3 Association between the BRI and COPD risk

To further investigate the relationship between the BRI and COPD, three multivariable regression models were assessed (Table 3). In Model 1, which did not adjust for covariates, each 1-unit increase in the BRI was associated with a 6.4% increased risk of COPD (OR = 1.064, 95% CI: 1.040–1.088, *p* < 0.001). In Model 2, which adjusted for covariates including age, sex, race, marital status, poverty-to-income ratio (PIR), education, hypertension, smoking status, alcohol use, DM, CKD, and CVD, the odds ratio (OR) for the BRI and COPD was 1.059 (95% CI: 1.032–1.087, *p* < 0.001). In Model 3, which included all covariates, the OR for BRI was further adjusted to 1.051 (95% CI: 1.022–1.080, *p* < 0.001). Furthermore, with increasing BRI, the risk of COPD continued to rise significantly (*p* for trend <0.005).

**Table 3:** Multivariable logistic regression models for the association between the BRI and COPD.

Model	Crude Model 1^a^		Model 2^b^		Model 3^c^	
	OR (95%CI)	*p*-value	OR (95%CI)	*p*-value	OR (95%CI)	*p*-value
BRI	1.064 (1.040–1.088)	<0.001	1.059 (1.032–1.087)	<0.001	1.051 (1.022–1.080)	<0.001
Categories						
Q1	Reference		Reference		Reference	
Q2	0.858 (0.733–1.003)	0.055	0.836 (0.709–0.985)	0.033	0.845 (0.716–0.998)	0.048
Q3	1.054 (0.907–1.225)	0.490	0.976 (0.831–1.147)	0.771	0.984 (0.835–1.161)	0.849
Q4	1.345 (1.167–1.553)	<0.001	1.242 (1.059–1.458)	0.008	1.206 (1.019–1.429)	0.030
*p* for trend^d^	<0.001		0.001		0.008	

a Without adjustment.

b Adjusted for age, sex, race, marital status, PIR, education, hypertension, smoking, alcohol, DM, CKD, and CVD.

c Adjusted for all variables.

d *p* for trend is calculated by converting the quartiles of the BRI into level variables, assigning values of 0, 1, 2, and 3, and then inputting the level variables into the regression model.

BRI: body roundness index.

**Figure 3:**
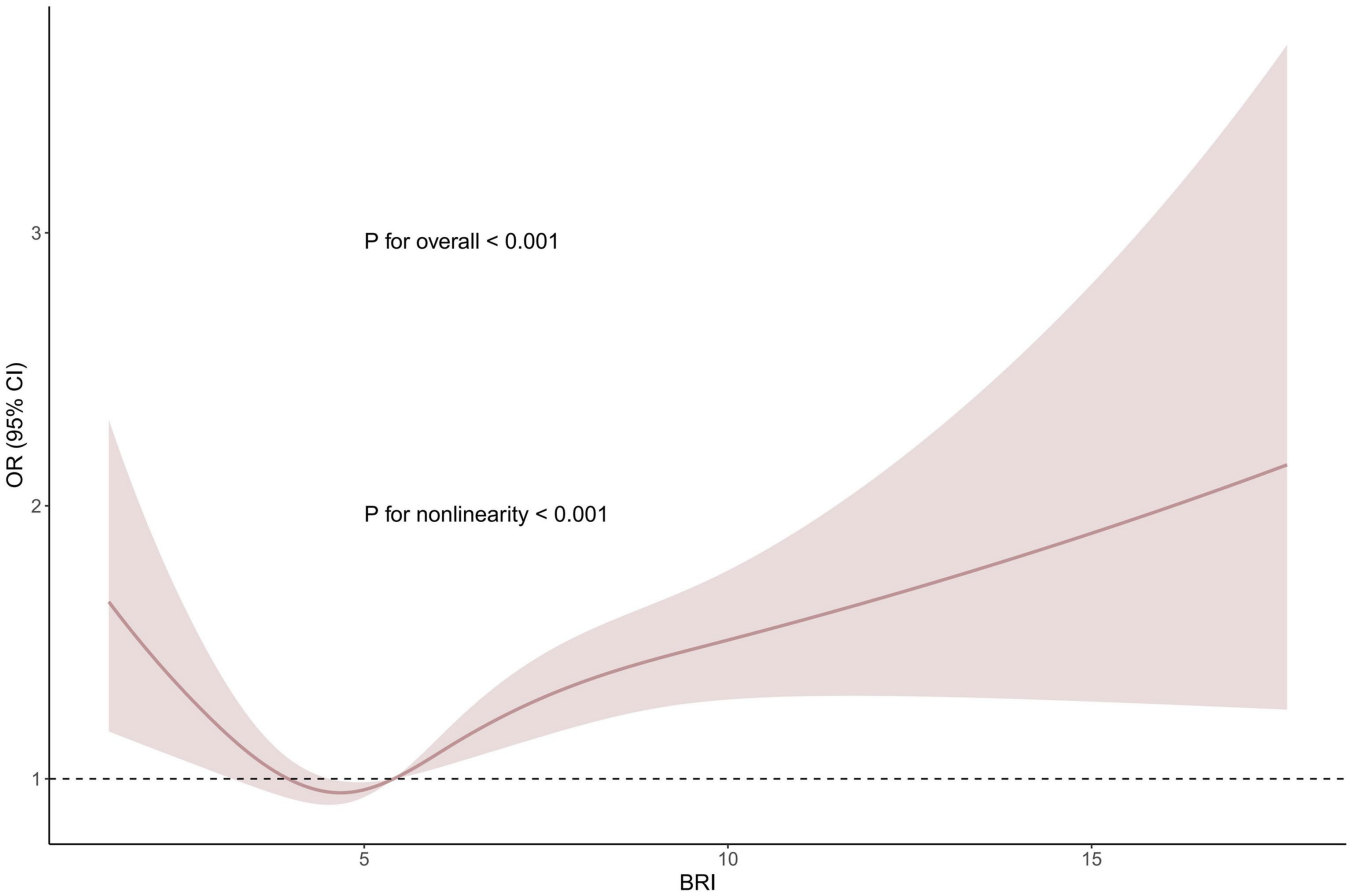
Smooth curve fitting (RCS analysis) between the BRI and COPD based on Model 3.

Based on Model 3, we performed a smooth curve fitting (RCS analysis) to further investigate the association between the BRI and COPD (Figure 3). The results revealed a significant non-linear relationship between the BRI and COPD (*p* for non-linearity <0.001). To further elucidate the nature of this relationship, we subsequently conducted a threshold effect analysis (Table 4). The analysis showed that, at the threshold point of BRI = 3.6583 (likelihood ratio <0.001), when the BRI was below this threshold, each one-unit increase in the BRI was associated with a 0.7586 decrease in the OR for COPD, indicating that an increase in the BRI was associated with a reduced COPD risk. However, once the BRI exceeded this threshold, an increase in the BRI was significantly associated with an elevated COPD risk (OR: 1.0756, 95% CI: 1.0426–1.1097, *p* < 0.001). These results highlight that, after surpassing a certain threshold, increases in the BRI significantly elevate the risk of COPD, further emphasizing the threshold-dependent effect of the BRI on COPD risk.

**Table 4:** Threshold effect analysis of the BRI on COPD using a two-piecewise logistic regression model.

Threshold effect analysis	COPD OR (95%CI)	*p*
Inflection points of the BRI	3.6583	
<3.6583	0.7586 (0.5660–1.0167)	0.0645
≥3.6583	1.0756 (1.0426–1.1097)	<0.001
*p* for likelihood test		<0.001

### 3.4 Sensitivity analysis

**Figure 4:**
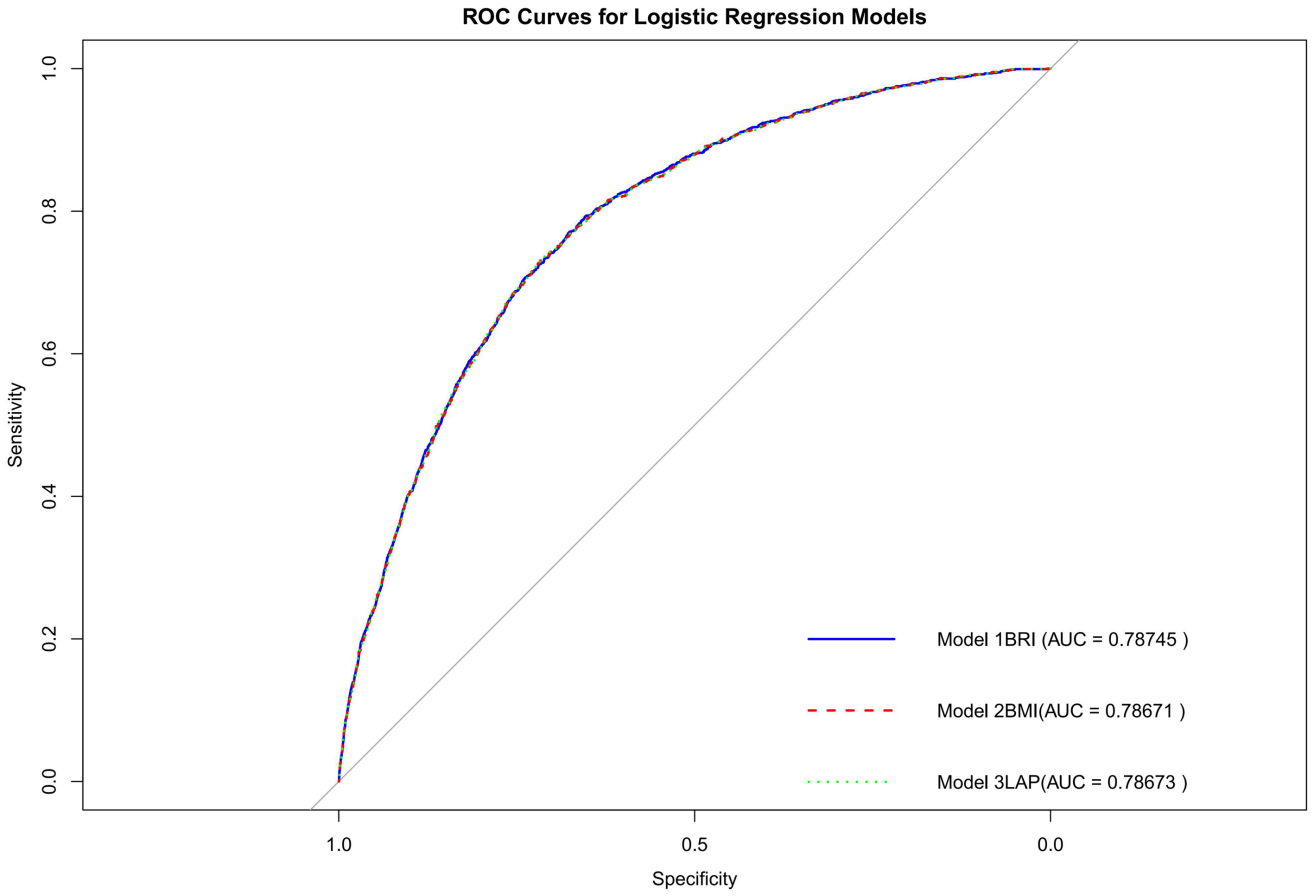
ROC curve.

Subsequently, we examined the association between the LAP and the BRI with the outcomes across models with increasing levels of adjustment (Table 5). In the crude model (Model 1), the LAP was significantly associated with the outcome (OR = 1.001, 95% CI: 1.000–1.002, *p* = 0.001). However, in the adjusted models, this association weakened. In Model 2, after adjusting for age, sex, race, marital status, PIR, education, hypertension, smoking, alcohol, DM, CKD, and CVD, LAP showed no significant association (OR = 1.000, 95% CI: 1.000–1.001, *p* = 0.482). Similarly, in Model 3, which included all variables, LAP remained non-significant (OR = 1.001, 95% CI: 1.000–1.001, *p* = 0.202). For the BMI, no significant association with COPD was found in Model 1 (unadjusted), Model 2 (adjusting for partial variables), or Model 3 (adjusting for all variables) (*p* > 0.05). However, after adjusting for all variables, COPD risk was relatively low in other BMI categories relative to the “underweight” group. The ROC curve (Figure 4) shows that the AUC of the BRI (0.78745) is higher than that of the LAP (0.78673) and the BMI (0.78671).

**Table 5:** Sensitivity analysis.

Model	Crude Model 1^a^		Model 2^b^		Model 3^c^	
	OR (95%CI)	*p*-value	OR (95%CI)	*p*-value	OR (95%CI)	*p*-value
LAP	1.001 (1.000–1.002)	0.001	1.000 (1.000–1.0001)	0.482	1.001 (1.000–1.001)	0.202
Categories						
Q1	Reference		Reference		Reference	
Q2	0.968 (0.832–1.127)	0.675	0.885 (0.754–1.037)	0.131	0.919 (0.782–1.079)	0.301
Q3	0.974 (0.837–1.133)	0.731	0.883 (0.752–1.036)	0.126	0.879 (0.745–1.038)	0.128
Q4	1.237 (1.071–1.429)	0.004	1.006 (0.857–1.181)	0.943	1.052 (0.888–1.247)	0.559
*p* for trend^d^	0.004		0.870		0.622	
BMI	0.996 (0.988–1.004)	0.327	1.007 (0.997–1.016)	0.156	1.004 (0.994–1.013)	0.477
Categories						
Underweight	Reference		Reference		Reference	
Normal	0.333 (0.240–0.471)	<0.001	0.486 (0.342–0.702)	<0.001	0.509 (0.357–0.741)	<0.001
Overweight	0.292 (0.212–0.410)	<0.001	0.459 (0.326–0.660)	<0.001	0.490 (0.344–0.711)	<0.001
Obese	0.360 (0.257–0.512)	<0.001	0.625 (0.433–0.917)	0.014	0.622 (0.425–0.924)	0.016
*p* for trend	0.074		0.554		0.807	
Obese						
BRI^e^	1.108 (1.071–1.145)	<0.001	1.086 (1.046–1.128)	*p* < 0.001	1.079 (1.038–1.122)	*p* < 0.001
Categories						
Q1	Reference		Reference		Reference	
Q2	0.920 (0.777–1.090)	0.333	0.879 (0.738–1.048)	0.151	0.882 (0.739–1.053)	0.164
Q3	1.131 (0.962–1.331)	0.137	1.032 (0.869–1.227)	0.717	1.032 (0.866–1.230)	0.729
Q4	1.412 (1.200–1.663)	<0.001	1.277 (1.069–1.526)	0.007	1.247 (1.036–1.502)	0.020
*p* for trend	<0.001		0.001		0.004	

a Without adjustment.

b Adjusted age, sex, race, marital status, PIR, education, hypertension, smoking, alcohol, DM, CKD, and CVD.

c Adjusted for all variables.

d *p* for trend is calculated by converting the quartiles of BRI into level variables, assigning values of 0, 1, 2, and 3, and then inputting the level variables into the regression model.

e BRI was calculated after removing the outliers of 5% before and after to ensure the stability and reliability of the results.

Excluding the top and bottom 5% of extreme BRI values (see Table 5), the analysis showed that the BRI remained significantly associated with COPD outcomes in the multivariable logistic regression model (*p* < 0.05). Subsequently, in Model 3, BMI, LAP, and both BMI and LAP were sequentially added (Supplementary Table 1). The results indicated that the BRI exhibited a significant independent effect. Moreover, after adjusting for the number of cigarettes smoked (Supplementary Table 2), the BRI remained significantly associated with COPD (OR = 1.048, 95% CI: 1.019–1.078, *p* = 0.001).

Finally, after reconfirming the COPD diagnosis using Forced Expiratory Volume in 1 second (FEV1) and Forced Vital Capacity (FVC), the analysis revealed a non-linear relationship between the BRI and COPD (*p* for non-linearity <0.001, Supplementary Figure 1). After adjusting for all variables, the analysis showed that the trend of the association between the BRI and COPD remained consistent (Supplementary Table 3).

### 3.5 Subgroup analysis

Further subgroup analysis and interaction tests (Table 6) revealed that, in most of the examined subgroups, higher levels of the BRI were significantly associated with increased COPD prevalence. Although the association between the BRI and COPD risk was more pronounced in certain specific populations, the interaction effect *p*-values for most subgroups did not show significant differences. Interestingly, a significant interaction between the BRI and sex was observed (interaction *p* = 0.045). In male patients, the BRI was significantly positively correlated with COPD risk (OR = 1.077, 95% CI: 1.034–1.12, *p* < 0.001), while this relationship was not significant in female patients. This suggests that the male population may be at a higher risk of COPD.

**Table 6:** Subgroup stratified analysis of the BRI and COPD with adjustments from Model 3.

Variables	OR	95%CI	*p* value	*p* for interaction
Age				0.573
<65	1.045	(1.007, 1.085)	0.019	
>65	1.038	(0.994, 1.083)	0.092	
Sex				0.045
Female	1.031	(0.991, 1.073)	0.131	
Male	1.077	(1.034, 1.12)	<0.001	
Race				0.087
Non-Hispanic White	1.027	(0.991, 1.064)	0.148	
Non-Hispanic Black	1.022	(0.958, 1.088)	0.495	
Other Hispanic	1.106	(0.975, 1.25)	0.11	
Mexican American	1.169	(1.041, 1.306)	0.007	
Other races—including multi-racial	1.205	(1.064, 1.361)	0.003	
Marital				0.964
Married	1.166	(1.06, 1.278)	0.001	
Never married	1.029	(0.988, 1.071)	0.165	
Divorced	1.023	(0.955, 1.094)	0.512	
Widowed	1.035	(0.962, 1.112)	0.352	
Separated	1.17	(1.022, 1.341)	0.023	
Living with partner	1.01	(0.87, 1.16)	0.887	
PIR				0.17
<1	1.072	(1.012, 1.134)	0.017	
>1	1.042	(1.009, 1.076)	0.012	
Education				0.343
Less than 9th grade	1.06	(0.972, 1.154)	0.181	
9–11th grade (includes 12th grade with no diploma)	1.059	(0.995, 1.126)	0.069	
High school graduate/GED or equivalent	1.031	(0.973, 1.091)	0.295	
College graduate or above	1.049	(0.97, 1.133)	0.225	
Some college or AA degree	1.053	(1.001, 1.108)	0.044	
Hypertension				0.223
No	0.994	(0.943, 1.046)	0.81	
Yes	1.068	(1.033, 1.104)	<0.001	
Smoke				0.359
Now	1.048	(0.98, 1.118)	0.161	
Former	1.046	(0.996, 1.098)	0.072	
Never	1.05	(1.008, 1.093)	0.018	
Alcohol				0.541
Former	1.043	(0.939, 1.153)	0.425	
Never	1.07	(1.018, 1.124)	0.007	
Mild	1.03	(0.979, 1.082)	0.251	
Moderate	0.953	(0.874, 1.036)	0.27	
Heavy	1.138	(1.06, 1.22)	<0.001	
DM				0.007
No	1.024	(0.989, 1.059)	0.185	
Yes	1.114	(1.06, 1.169)	<0.001	
CKD				0.597
No	1.046	(1.01, 1.083)	0.011	
Yes	1.051	(1.002, 1.102)	0.041	
CVD				0.046
No	1.038	(1.003, 1.074)	0.031	
Yes	1.075	(1.022, 1.13)	0.005	
Alt				0.829
<40	1.051	(1.021, 1.082)	0.001	
>40	1.074	(0.967, 1.188)	0.174	
Albumin (g.L)				0.262
35–50	1.069	(1.04, 1.099)	<0.001	
<35 or >50	0.93	(0.793, 1.081)	0.355	
Creatinine (mg.dl)				0.035
<1.4	1.042	(1.011, 1.072)	0.006	
>1.4	1.158	(1.046, 1.281)	0.004	
Uric acid (mg.dl)				0.035
<7.2	1.025	(0.995, 1.056)	0.105	
>7.2	1.139	(1.064, 1.218)	<0.001	
Refrige total cholesterol (mg.dl)				0.825
<200	1.047	(1.01, 1.085)	0.011	
>200	1.059	(1.012, 1.107)	0.012	
Monocyte number (1000cells.ul)				0.707
<0.8	1.044	(1.011, 1.077)	0.008	
>0.8	1.071	(1.009, 1.136)	0.023	
Segmented neutrophil number (1000cells.ul)				0.009
<7	1.044	(1.013, 1.076)	0.004	
>7	1.083	(0.999, 1.174)	0.052	
Eosinophil number (1,000 cells.ul)				0.023
<0.5	1.064	(1.033, 1.095)	<0.001	
>0.5	0.929	(0.846, 1.017)	0.115	
Basophil number (1,000 cells.ul)				0.985
<0.1	1.045	(1.004, 1.086)	0.029	
>0.1	1.048	(1.007, 1.091)	0.02	
Cancer				0.171
No	1.055	(1.023, 1.088)	0.001	
Yes	1.022	(0.955, 1.091)	0.527	
Platelet				0.597
150–450	1.045	(1.015, 1.075)	0.003	
<150 or >450	1.078	(0.96, 1.206)	0.194	
Cotinine (ng.ml)				0.113
<10	1.054	(1.017, 1.092)	0.004	
>10	1.034	(0.988, 1.081)	0.144	

Moreover, significant interaction effects between the BRI and DM, CVD, creatinine, uric acid, segmented neutrophil number, and eosinophil number were observed (interaction *p* < 0.05). Specifically, in populations with DM (OR: 1.114, 95% CI: 1.06–1.169, *p* < 0.001), creatinine >1.4 mg/dL (OR: 1.158, 95% CI: 1.046–1.281, *p* = 0.004), uric acid > 7.2 mg/dL (OR: 1.139, 95% CI: 1.064–1.218, *p* < 0.001), and eosinophil number <0.5 × 10^3^/μl (OR: 1.064, 95% CI: 1.033–1.095, *p* < 0.001), significant positive correlations between the BRI and COPD risk were observed, indicating that these populations may be at a higher risk of COPD.

## 4 Discussion

This study conducted a cross-sectional analysis of data from 24,873 individuals aged 40 years and above, collected from the NHANES between 1999 and 2018. The results revealed a significant positive correlation between the BRI and COPD. Specifically, after adjusting for all covariates in Model 3, each 1-unit increase in BRI was associated with a 5.1% increase in the risk of developing COPD. Moreover, the incidence of COPD was significantly higher in the high-BRI group compared to the low-BRI group, with an overall trend being significant (P for trend = 0.008). RCS analysis further revealed a U-shaped non-linear relationship between the BRI and COPD. Threshold effect analysis identified the optimal cutoff value of BRI as 3.6583, and sensitivity analysis further confirmed the stability and consistency of the study’s findings.

The BRI, as a composite index assessing height, waist circumference, and weight, more accurately reflects abdominal fat distribution, particularly visceral fat accumulation. Compared to traditional BMI, the BRI shows higher sensitivity in evaluating health risks related to abdominal fat, especially the accumulation of visceral fat, which plays an important role in the onset and progression of COPD (23, 24). Visceral fat exacerbates airway inflammation by secreting pro-inflammatory factors such as tumor necrosis factor-alpha (TNF-*α*), interleukin-6 (IL-6), and C-reactive protein (CRP), thereby playing a key role in the pathological process of COPD (14, 25–27). The release of these inflammatory mediators promotes chronic inflammation and airway remodeling, leading to a gradual decline in lung function (28). Additionally, visceral fat accumulation is closely associated with increased oxidative stress levels. Oxidative stress damages lung cells, destroys alveolar structures, exacerbates pulmonary dysfunction, and impairs the lung repair mechanism, leading to irreversible lung damage in COPD patients and exacerbating airway remodeling (29–31). Excessive abdominal fat accumulation may also elevate the diaphragm, reduce chest cavity volume, restrict lung expansion, and increase the burden on respiratory muscles, leading to dyspnea and further worsening COPD symptoms, thereby creating a vicious cycle (32). Simultaneously, abdominal fat accumulation may affect hormone levels such as cortisol and estrogen. Elevated cortisol inhibits immune system function and increases infection risk, while changes in estrogen levels may modulate pulmonary inflammatory responses and repair mechanisms (33–35). The increase in abdominal fat may also cause autonomic nervous system dysfunction, affecting airway contraction and relaxation, enhancing airway hyperreactivity, and leading to bronchospasm and dyspnea, which exacerbates clinical symptoms of COPD (36, 37). The BRI can sensitively reflect the accumulation of abdominal fat and reveal its close association with metabolic syndrome, including hypertension, hyperglycemia, and hyperlipidemia (38).

Compared to the LAP index, the BRI shows higher accuracy in predicting COPD risk. This is primarily because the BRI more precisely reflects the accumulation of visceral fat, which is considered a key pathological factor in COPD (4). Through BRI assessment, we can more effectively predict the risk of COPD. While LAP also reflects fat accumulation and metabolic abnormalities, its relationship with COPD is more focused on the impact of metabolic dysregulation and lipid accumulation, and its mechanistic depth and comprehensiveness may not match that of the BRI (4).

Subgroup analysis results indicate significant differences in the role of gender, diabetes, CVD, and other biomarkers (such as creatinine, uric acid, and eosinophil counts) in the risk of COPD. Gender differences play an important role in the occurrence of COPD, with males having a significantly higher risk than females. This difference is closely related to disparities in lung function, airway structure, and immune responses (39). Male airway structures may be more vulnerable to damage from tobacco smoke and air pollution, and males generally have higher smoking rates, with smoking still being the primary risk factor for COPD. Although smoking rates among females are increasing, male smokers are more likely to develop COPD due to prolonged smoking (39–41). Furthermore, the anti-inflammatory effects of estrogen in women may represent a key mechanism explaining the gender differences observed between the BRI and COPD. Estrogen regulates immune cell function by activating estrogen receptors (ER*α* and ERβ) and inhibits the production of pro-inflammatory cytokines, such as IL-6 and TNF-*α*, thereby reducing chronic inflammation (42, 43). This immunosuppressive effect helps maintain a balanced immune response, allowing women to exhibit milder immune reactions when exposed to environmental stimuli (such as smoking or air pollution), which may consequently reduce the incidence and progression of COPD. Estrogen also modulates dendritic cells (DCs) and macrophages, promoting the production of anti-inflammatory cytokines, further mitigating inflammation (44–46). Furthermore, estrogen explains gender differences by influencing immune cell function. Studies have shown that dendritic cells in women are more efficient in activating immune responses, particularly in producing type I interferons (such as IFN-α and IFN-*β*), compared to men (47–49). This enhanced ability results in a more tempered immune response in women when facing pathogens or environmental triggers, whereas men’s immune systems are more prone to overreaction. This may explain why the relationship between the BRI and COPD is more pronounced in men (50). Estrogen may also play an important role in lung tissue through its receptors (ERα, ERβ, and GPER). Research indicates that in female COPD patients, the expression of estrogen receptors and metabolic genes in lung tissue is increased, suggesting that estrogen levels may promote the progression of COPD by influencing pulmonary immune responses (51–53). Estrogen further exacerbates COPD pathology by regulating the expression of aromatase (CYP19A1) and 17β-hydroxysteroid dehydrogenase 1 (HSD17B1) (54).

In contrast, the relationship between testosterone levels and COPD in men is more complex. Some studies suggest that testosterone levels are positively correlated with lung function, particularly FEV1, such that higher testosterone levels are associated with better lung function (55–57). Testosterone may, to some extent, support lung tissue structure and function, including promoting alveolar development and maintaining airway integrity, thereby helping men sustain better lung function (55). Additionally, gender differences in fat distribution may also influence the risk of developing COPD. Women typically exhibit a “pear-shaped” fat distribution, with fat predominantly accumulating in the hips and thighs, while men more often show an “apple-shaped” distribution, with increased abdominal fat. Abdominal fat, particularly visceral fat, is closely associated with the onset and progression of COPD (58–60). Therefore, the fat distribution characteristics in women may result in a lower COPD risk at the same BRI level compared to men. Diabetes is a pro-inflammatory condition, and prolonged high blood glucose levels lead to chronic low-grade systemic inflammation, which not only exacerbates airway damage but may also promote the onset and progression of COPD (41). High blood glucose states promote chronic airway inflammation, leading to the gradual decline of lung function (61). For COPD patients with comorbid cardiovascular diseases, hypertension, coronary artery disease, and other issues often exacerbate the burden on the heart, reducing cardiopulmonary function and accelerating COPD progression (62, 63). Furthermore, abnormalities in biomarkers such as creatinine, uric acid, and eosinophil counts significantly impact COPD risk. These biomarkers often reflect underlying health issues across multiple physiological systems, and abnormal levels of these biomarkers in patients with cardiovascular diseases and chronic kidney diseases may signal an increased risk of COPD (64–67).

BRI, as an emerging body size assessment tool, compared with the traditional body mass index (BMI) and waist–hip ratio (LAP), can more accurately reflect the body size characteristics of individuals, especially in the assessment of COPD risk. Our results showed a significant association between the BRI and the occurrence and progression of COPD, suggesting that BRI is expected to become an important tool for early screening of COPD. The assessment combined with BRI can provide more accurate COPD risk prediction for high-risk groups, especially in those high-risk individuals who have not yet developed obvious clinical symptoms (such as long-term smokers or patients with family history).

### 4.1 Strengths and limitations

The strength of this study lies in the use of the large-scale NHANES dataset to systematically analyze the relationship between the BRI and COPD. To the best of our knowledge, this is the first study to assess the association between the BRI and COPD risk using NHANES data from 1999 to 2018. This study has several strengths: first, the large sample size not only enhances the accuracy of statistical analysis but also provides sufficient data to investigate the correlation between the BRI and COPD in different subgroups, revealing how this association varies according to demographic factors (including gender, age groups, and smoking habits). However, this study has its limitations. Due to the cross-sectional nature of the study, establishing a clear causal relationship between variables remains challenging. This means that, although an association between the BRI and COPD has been observed, we cannot determine whether changes in the BRI directly lead to an increased risk of COPD. Second, the diagnosis of COPD relies on self-reported symptoms, lacking objective clinical diagnostic criteria, which may lead to recall bias or misclassification, thereby affecting the accuracy of the results. Finally, smoking, as a major etiological factor of COPD, although this study includes serum cotinine and considers smoking quantity in the sensitivity analysis, still cannot fully reflect the cumulative exposure to smoking (such as pack-years). Therefore, this study fails to further explore the specific relationship between smoking exposure and COPD, which is also an important limitation of the study. Future studies should consider adopting a longitudinal design and strengthening the comprehensiveness of data collection, which will help to better understand how BRI affects the occurrence and progression of chronic obstructive pulmonary disease.

## 5 Conclusion

This study identified a significant non-linear relationship between the BRI and the risk of COPD. Higher levels of the BRI were associated with an increased risk of COPD, with each 1-unit increase in the BRI corresponding to a 5.1% increase in the risk of developing COPD. Moreover, the association was non-linear, with a threshold effect identified at a BRI value of 3.6583, where the risk of COPD increased significantly beyond this threshold. These findings suggest that the BRI is a valuable and more accurate indicator of abdominal fat distribution compared to traditional measures, such as the LAP, for assessing COPD risk. The results support the potential of the BRI as a predictive tool for COPD, offering an important foundation for early diagnosis and targeted interventions in clinical practice.

## Data Availability

Publicly available datasets were analyzed in this study. This data can be found at: data repository: [National Health and Nutrition Examination Survey (NHANES)] direct link to data: https://www.cdc.gov/nchs/about/index.html.
