# Supplementary material for: Correlation between the body roundness index and chronic obstructive pulmonary disease: a cross-sectional analysis
Source: Front Nutr. 2025 Aug 25;12:1624617. doi: 10.3389/fnut.2025.1624617 (PMC12414733; doi:10.3389/fnut.2025.1624617)
Supplement: Supplementary file 1 [file Table_1.docx]

| **Supplementary Table 1** | | | | | | |
| --- | --- | --- | --- | --- | --- | --- |
|  | **Model1^a^** | | **Model2^b^** | | **Model3^c^** | |
|  | **OR (95%CI)** | **P value** | **OR (95%CI)** | **P value** | **OR (95%CI)** | **P value** |
| **BRI** | **1.055(1.023-1.088)** | **0.001** | **1.301(1.211-1.398)** | **<0.001** | **1.305(1.213-1.403)** | **<0.001** |
| **Categories** |  |  |  |  |  |  |
| **Q1** | **Reference** |  | **Reference** |  | **Reference** |  |
| **Q2** | **0.847(0.716-1.001)** | **0.052** | **0.922(0.771-1.103)** | **0.376** | **0.921(0.769-1.102)** | **0.367** |
| **Q3** | **0.987(0.832-10171)** | **0.88** | **1.150(0.940-1.409)** | **0.175** | **1.146(0.935-1.406)** | **0.19** |
| **Q4** | **1.212(1.007-1.460)** | **0.042** | **1.617(1.229-2.130)** | **0.001** | **1.608(1.218-2.126)** | **0.001** |
| **P for trend** | **0.015** | | **0.001** | | **0.001** | |
| ***a****: The model 3 was expanded by including LAP (Waist-to-Hip Ratio) as an additional predictor variable.*  *b: The model 3 was expanded by including BMI (Body Mass Index) as an additional predictor variable.*  *c: The model 3 was expanded by including both LAP (Waist-to-Hip Ratio) and BMI (Body Mass Index) as predictor variables.* | | | | | | |

| **Supplementary Table 2** | | |
| --- | --- | --- |
|  | **OR (95%CI)** | **P value** |
| **BRI** | **1.048(1.019-1.078)** | **0.001** |
| **Categories** |  |  |
| **Q1** | **Reference** |  |
| **Q2** | **0.869(0.734-1.029)** | **0.105** |
| **Q3** | **0.981(0.829-1.161)** | **0.824** |
| **Q4** | **1.198(1.008-1.423)** | **0.04** |
| **P for trend** | **0.015** | |
| *The model 3 was expanded by including smoke number as an additional predictor variable.* | | |

| **Supplementary Table 3** | | |
| --- | --- | --- |
|  | **OR (95%CI)** | **P value** |
| **BRI** | **1.000(0.977-1.024)** | **0.97** |
| **Categories** |  |  |
| **Q1** | **Reference** |  |
| **Q2** | **0.825(0.725-0.939)** | **0.004** |
| **Q3** | **0.808(0.707-0.923)** | **0.002** |
| **Q4** | **0.911(0.792-1.047)** | **0.190** |
| **P for trend** | **0.178** | |
| *The diagnostic criteria of COPD are based on two ways: one is the self-reported data of COPD diagnosed by doctors; The second is to pass the standard of FEV1/FVC ratio less than 0.7. The remaining individuals without COPD were further studied in this analysis.* | | |

| 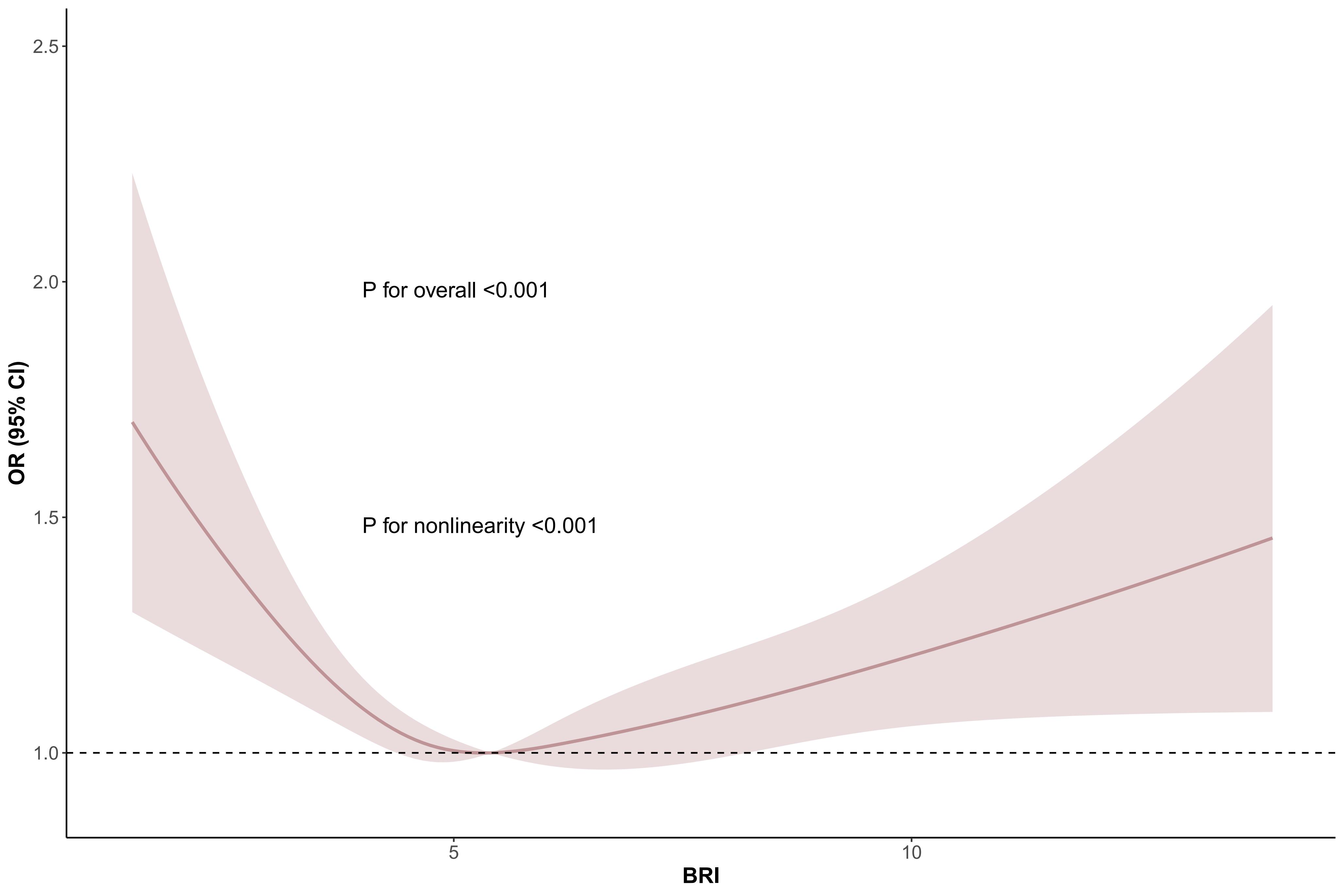  **Supplementary Figure 1**  *RCS analysis shows that there is a significant non-linear relationship between BRI and COPD based on two diagnostic methods of COPD, and it shows a trend of first decreasing and then increasing, which is consistent with the conclusion.* |
| --- |
